# Diabetes SPECIAL (Students Providing Education on Chronic Illness and Lifestyle): a novel preclinical medical student elective

**DOI:** 10.1007/s40037-020-00641-w

**Published:** 2020-12-21

**Authors:** Sarah E. Myers, Nicholas R. Bender, Marina A. Seidel, Ruth S. Weinstock

**Affiliations:** Department of Medicine: Division of Endocrinology, Diabetes and Metabolism, SUNY Upstate Medical University, Syracuse, NY USA

**Keywords:** Medical student education, Diabetes education, Preclinical electives

## Abstract

**Background:**

Traditional medical student curricula limit substantial clinical experiences until the third and fourth years of medical school. This delay in valuable experiences hinders the ability of some medical students to choose a specialty to pursue, delays the formation of meaningful longitudinal mentorship relationships, and limits the development of important clinical acumen. Furthermore, the use of medical students in preclinical years may help to improve patient care and outcomes.

**Approach:**

The novel preclinical Diabetes SPECIAL (Students Providing Education on Chronic Illness and Lifestyle) elective was designed to introduce first year medical students to the field of endocrinology, promote the development of a professional identity, improve medical student communication skills, and raise awareness of the complexities of managing patients living with diabetes mellitus. Furthermore, and novel to this experience, was to measure the impact of this elective on patient outcomes.

**Evaluation:**

Students attended patient appointments, communicated with their assigned patients regularly, relayed important health information to the attending endocrinologist, and attended monthly didactic sessions. The elective outcomes were evaluated via completed surveys by patients, students, and attending physicians as well as medical record review for pre- and post-elective hemoglobin A1C levels.

**Reflection:**

Students, faculty, and patients who participated in this elective generally reported having a positive experience. Seven out of 10 patients had a reduction in their hemoglobin A1C levels. The outcomes from the pilot of this novel preclinical elective support the importance of early clinical exposure in medical student training and highlight potential positive impacts on both medical student education and patient outcomes.

## Background and need for innovation

In the traditional 4‑year medical student curriculum, students primarily study basic sciences for the first 2 years with minimal clinical exposure prior to transitioning to clinical clerkships for the last 2 years of training [1]. While a strong basic science foundation is essential to a medical trainee’s education, several challenges arise when the majority of clinical exposures are restricted to the final 2 years of medical school.

The process of becoming a physician not only requires the acquisition of medical knowledge and clinical problem-solving skills, but also the development of a professional identity [2]. Several reports have examined psychological and sociological theories regarding how a medical student’s professional identity is developed [3–5]. The conceptual change theory, for example, proposes that when individuals experience cognitive conflict, or cognitive disequilibrium, they must reconcile this disturbance by creating new concepts and ways of thinking that are beneficial to oneself [3].

Using the conceptual change theory as a model, Kay et al. sought to identify the experiences in medical school that introduced cognitive disequilibrium. Importantly, four experiences were identified, namely: the transition from undergraduate student to medical student, clinical experiences in the preclinical years, exposure to the business aspect of medicine, and exposure to physicians in clinical practice [3]. By targeting these vulnerable periods with impactful experiences, researchers suggested that educators may see further development of the professional identity.

Aside from professional identity development, a positive impact on learning and clinical aptitude may also occur as a result of these experiences. Researchers reported a shift in students’ motivation for studying after partaking in preclinical experiences. Students reported wanting to perform well on examinations to provide high-quality patient care rather than simply to achieve high grades [3]. Additionally, in previously implemented electives, students reported feeling more confident once in clinical clerkships and have been reported to perform superiorly, resulting in more honors grades, when compared with classmates who did not participate [6, 7].

The implementation of preclinical electives has increased in previous years, most notably within surgical specialties [6–12]. Increasingly competitive residency application cycles, concern about lifestyle, and lack of exposure to potential career fields likely contributed to the recognized need for early exposure in clinical training. By delaying such exposures, students are more likely to choose their specialty later in their medical training and may be unable to complete meaningful research projects, develop longitudinal relationships with mentors, and prepare strong residency applications [12]. Thus, aside from fostering the development of a student’s professional identity, early exposure to medical and surgical specialties via preclinical electives may increase interest in fields not commonly experienced by medical students, increase the proficiency of medical students on core clinical rotations, and allow students to better prepare strong residency applications.

## Goal of innovation

The benefits of better preparing students for residency, increasing proficiency on clinical rotations, and fostering the development of a professional identity would likely be seen across all disciplines, including internal medicine and associated sub-specialties. In addition to gaining exposure to internal medicine and related sub-specialties, students would begin to appreciate the intricacies of managing patients with complex medical problems, improve their communication skills, and begin to understand the importance of interdisciplinary patient care. Lastly, there is a potential for medical students to contribute to better patient outcomes as they are given a distinct role and responsibility during their preclinical electives to be actively engaged with and advocate for their patients. To our knowledge, none of the developed preclinical electives have examined the potential clinical benefit that such experiences may have on patient care and outcomes.

Here we describe a novel preclinical medical student elective that was developed to expose students to the field of endocrinology, to improve medical student knowledge surrounding the complexities of managing patients living with diabetes mellitus, to promote the development of a professional identity, and to improve glycemic control as a result of the partnership established between a medical student and their patient.

## Steps taken for development and implementation of innovation

The Diabetes SPECIAL elective was developed by three second year medical students and an endocrinologist. Given the recognized need for integration of clinical experiences into the preclinical years, the elective was offered to first year medical students. The didactic and clinical portions of the elective were adapted from previously implemented preclinical electives including specific preclinical experiences where medical students functioned as patient navigators for patients living with a chronic disease [1, 13, 14].

For this pilot study, 10 first year medical students were enrolled and 10 attending endocrinologists volunteered to mentor one student each for the duration of the semester-long course. Attending endocrinologists identified an established patient whom they felt would benefit from having additional support from a medical student. After patients were identified and agreed to participate in the experience, medical students were assigned a patient and an attending endocrinologist with whom to work.

Students were expected to communicate with their patient at least once weekly focusing on encouragement of adherence to their individualized treatment plans. Students kept a record of such interactions and were asked to identify barriers to care, working with their mentors to help alleviate these problems. Additionally, they collected blood glucose data [from a continuous glucose monitor and/or meter] from their patients and sent this information to their endocrinologists for evaluation and recommendations for changes. Students were encouraged to attend routine medical appointments (i.e. diabetes, podiatry, cardiology, nutrition, wound care) with their patients.

In addition to participating in direct patient care, students attended monthly didactic sessions presented by diabetes team members, including introduction to diabetes and language of diabetes (endocrinologist), nutrition (dietitian), psychosocial issues and motivational interviewing (health psychologist), and physical activity (physical therapist/certified diabetes educator) to supplement their knowledge of comprehensive diabetes care. Students also discussed their cases and were able to troubleshoot problems or concerns amongst their peers and group leader.

## Outcomes of innovation/evaluation of innovation

Grading for the course was pass/fail based on participation in didactic sessions, completed logs of patient interactions, routine communication with patients, and completion of required course surveys. All students completed the post-course surveys and generally reported a satisfactory experience while participating in the elective. Patients and faculty members also completed surveys detailing their experiences as participants in the elective. The majority of patients returned the post-course surveys and of those that did, all reported a positive experience. Many patients reported that simply knowing someone would hold them accountable helped them to adhere to their treatment plans.

Upon completion of the elective, the majority of attending endocrinologists reported benefits for both their patient and medical student. Many reported they would consider being a mentor again and hoped to see the elective continue. However, there were two reports of minimal interactions with students and a sub-optimal experience due to lack of regular communication. Clearer expectations regarding communication will need to be established in future elective offerings.

To objectively measure the impact of the elective on diabetes care, pre- and post-elective hemoglobin A1c (HbA1c) results were obtained from the electronic medical records. Seven out of 10 patients showed a reduction in HbA1c (−6.3–0.6%), two patients had no change (HbA1c ± 0.1%) and one patient had an increase in HbA1c (initial HbA1c 7.1%). The small number of participants in this elective precluded further meaningful statistical analyses of these results. The greatest improvements in HbA1c levels were associated with the highest initial HbA1c levels. For example, in the patients with the four highest initial HbA1c levels (14.9%, 14%, 11.5%, and 9.1%), the reduction in HbA1c levels was 6.3%, 1.8%, 2.7%, and 1.9% respectively. Decreases of this magnitude were generally not seen in patients with lower initial HbA1c levels.

Many factors could explain the observed improvement in HbA1c levels during the elective including the expected impact of standard diabetes management and changes in treatment regimens that occurred as a result of routine care. However, for several of the patients who experienced improvements in HbA1c, documentation in the electronic medical records clearly denoted the impact of medical student involvement. Examples of this are noted below.

One patient was having severe post-prandial hypoglycemic episodes. According to the electronic medical records, due to frequent communication with her medical student and endocrinologist, her pump settings were adjusted three times between scheduled appointments with noted reductions in hypoglycemic episodes. This same patient, with the encouragement of her medical student, began testing her blood glucose levels more frequently, enabling her to qualify (per her insurance guidelines) for a continuous glucose monitor. With increased testing and prompt treatment changes, her HbA1c decreased by 1.9% over the course of the elective. She described in her post-course survey that participating in the elective “changed my life.”

Another patient had significant improvement in his glycemic control. When the elective began, the patient, who lives alone and is disabled, was infrequently testing his blood glucose levels. The student worked to help this patient obtain a continuous glucose monitor by aiding in the insurance authorization process. When he started working with his medical student, his HbA1c was 11.5% and had been above 9.0% for all recorded readings in the 2 years prior to this elective. His mean glucose was 314 ± 103 mg/dL, time in range was 7%, and 93% of his readings were >180 mg/dL. Nearly a year later the patient is wearing his continuous glucose monitor all the time and overall has improved his diabetes self-management. His mean glucose is now 154 ± 41 mg/dL, time in range is 75%, and only 22% of his readings are >180 mg/dL. The patient told his endocrinologist that the relationship he developed and maintained with his medical student has changed his life. While many factors may have contributed to improvement in glycemic control for the participating patients, student involvement clearly positively impacted the care of at least several patients.

## Critical reflection

Preclinical medical student electives are becoming increasingly popular in medical schools across the United States [1]. To our knowledge, the Diabetes SPECIAL elective is the first preclinical medical student elective in which medical students partnered directly with an attending endocrinologist and a patient living with diabetes mellitus in a role designed to directly improve diabetes management. This close partnership allowed for rapid treatment changes when needed to improve patient care.

This report has several limitations. A major limitation was the small number of participants which precluded meaningful statistical analyses of reductions seen in HbA1c levels. Additionally, the reductions seen in HbA1c levels could be attributed to factors outside of medical student involvement. The small number of participants, single pre-and post-intervention analysis of HbA1c levels, and lack of a control group are further limitations. Additionally, the benefits for patients may extend beyond the duration of the elective, and longer standardized follow-up periods may be helpful to confirm this hypothesis.

In subsequent offerings, students, patients, and mentors need to be fully aware of the expectations of the elective and must be willing to communicate regularly with one another. Finally, while this elective introduced students to the field of endocrinology, whether participation will lead to an increased interest in endocrinology or internal medicine as a career is unknown. This endpoint should be assessed in future offerings of the elective.

With evolving medical student curricula, early preclinical exposure through electives is becoming increasingly popular. While widespread implementation of electives such as this one may not be feasible for all preclinical students, outcomes of future preclinical electives must be reported in the literature to continue to understand the importance and potential benefits to both medical students and patients. By actively participating in patient care early in medical education, students begin to develop their professional identities and clinical acumen sooner, creating a framework of knowledge and skills that will be rapidly built upon in the clinical years. Lastly, the results reported in this pilot study suggest that the use of preclinical medical student electives may extend beyond the benefits for the medical student. While learning the intricacies of practicing medicine, preclinical medical students can actively participate in patient care by providing valuable support to patients, potentially improving their self-care and clinical outcomes.
